# Identification of Novel and Differentially Expressed MicroRNAs of Dairy Goat Mammary Gland Tissues Using Solexa Sequencing and Bioinformatics

**DOI:** 10.1371/journal.pone.0049463

**Published:** 2012-11-14

**Authors:** Zhibin Ji, Guizhi Wang, Zhijing Xie, Jianmin Wang, Chunlan Zhang, Fei Dong, Cunxian Chen

**Affiliations:** Shandong Provincial Key Laboratory of Animal Biotechnology and Disease Control and Prevention, College of Animal Science and Veterinary Medicine, Shandong Agricultural University, Taian, P.R. China; The Ohio State University, United States of America

## Abstract

MicroRNAs are small, noncoding RNA molecules that regulate gene expression at the post-transcriptional level and play an important role in various biological processes. Although most microRNAs expression profiles studies have been performed in humans or rodents, relatively limited knowledge also exists in other mammalian species. The identification of the full repertoire of microRNAs expressed in the lactating mammary gland of *Capra hircus* would significantly increase our understanding of the physiology of lactating mammary glands. In this study, two libraries were constructed using the lactating mammary gland tissues of Laoshan dairy goats (*Capra hircus*) during peak and late lactation. Solexa high-throughput sequencing technique and bioinformatics were used to determine the abundance and differential expression of the microRNAs between peak and late lactation. As a result, 19,044,002 and 7,385,833 clean reads were obtained, respectively, and 1,113 conserved known microRNAs and 31 potential novel microRNA candidates were identified. A total of 697 conserved microRNAs were significantly differentially expressed with a P-value<0.01, 272 microRNAs were up-regulated and 425 microRNAs were down-regulated during peak lactation. The results were validated using real-time quantitative RT-PCR. 762,557 annotated mRNA transcripts were predicted as putative target gene candidates. The GO annotation and KEGG pathway analysis suggested that differentially expressed microRNAs were involved in mammary gland physiology, including signal transduction, and cell-cell and cell-extracellular communications. This study provided the first global of the microRNA in *Capra hircus* and expanded the repertoire of microRNAs. Our results have great significance and value for the elucidation of complex regulatory networks between microRNAs and mRNAs and for the study of mammary gland physiology and lactation.

## Introduction

MicroRNAs (miRNAs) represent a class of small (∼22 nucleotides (nt) in length), endogenous, noncoding functional RNA molecules, which generally exist in eukaryotes and have spatiotemporal and tissue-specific expression patterns [1], [2]. It is estimated that miRNA genes might account for 2 to 5% of the total number of all mammalian genes and collectively regulate up to 60% of protein-coding genes [3], which makes miRNAs one of the most abundant classes of regulators in animals [4]. In mammals, miRNAs regulate protein expression via translation repression, or in some cases, via target mRNA degradation by incomplete complementary binding to the 3′-untranslated region (3′-UTR) [5], [6]. It has currently been established that miRNAs are involved in the regulation of well-defined developmental and cell-type specific processes, including cell proliferation, differentiation, programmed apoptosis and cell death [7], [8], morphogenesis of specific organs and the pathogenesis of human diseases [6], [9]. Recent studies have provided important clues in mammary gland development progress indicating that these small RNAs might have been a key factor for mammary gland development progress through their regulation of gene expression. Tanaka et al. [10] revealed that some miRNAs exhibit changes in their expression during mouse mammary gland epithelial cell differentiation. In particular, these authors observed that miRNA-101a plays important roles in mouse mammary development. Ucar et al. [11] showed that the miRNA-212/132 family is indispensable during mice mammary gland development, particularly for the regulation of epithelial duct outgrowth. In clinical research, the results of numerous studies have demonstrated that miRNAs function as tumor suppressor genes or oncogenes, and their expression was associated with breast tumorigenesis and carcinogenesis [12], [13]. The majority of the reported miRNA research has been focused on disease-related studies in either human or rodents, while the importance of miRNAs in other mammals need further be elucidated, and the essential roles of miRNAs in terms of post-transcriptional gene regulation in lactating mammary gland physiology and lactation of *Capra hircus* currently remains largely unknown.

Solexa high-throughput sequencing is a massively parallel sequencing platform, which are the most commonly used technique for miRNA expression profiling, especially for the species without available whole genome information [14], [15]. In recent years, following the development of sequencing technique and bioinformatics, a large number of miRNAs have been discovered and deposited in miRBase (Sanger miRNA database, http://www.mirbase.org/) or GEO (Gene Expression Omnibus, http://www.ncbi.nlm.nih.gov/geo/). Currently, 18,226 entries representing hairpin precursor miRNAs, expressing 21,643 mature miRNA products in 168 species have been identified and deposited in the public miRNA database miRBase (Release 18.0, November 2011) [16]. However, the number of miRNAs identified from domesticated ruminants, such as *Bos taurus* and *Ovis aries* (676 and 103, respectively), is far less than those identified from other mammals. Moreover, no miRNAs from *Capra hircus* have been deposited in the miRBase 18.0. In this study, we provided the first global of the miRNA expression profiles (miRNome) in *Capra hircus* and expanded the repertoire of miRNAs.

The mammary gland undergoes a cycle of cell division, differentiation, de-differentiation and cell death during its lactation process in adults, making it a well-established model organ for studying the molecular mechanism underlying lactation and mammary gland physiology [17]. The Laoshan dairy goat (*Capra hircus*), one of four excellent dairy goat breeds in China, is an ideal model organism for studying the molecular mechanisms underlying lactating mammary gland and biological progress. To gain further insight into the potential role of miRNAs in lactating mammary gland, two libraries were generated from lactating mammary gland tissues during different lactation periods (90 and 210 days postpartum, respectively). We integrated Solexa high-throughput sequencing technique and bioinformatics for sequencing and data processing to obtain the miRNAs expression profiles during the two lactation periods, identify the novel and differentially expressed miRNAs, define the regulatory network between miRNAs and mRNA, determine the regulatory mechanisms of miRNAs involved in the lactating mammary gland, and would significantly increase our understanding of the physiology of lactating mammary gland.

## Materials and Methods

### Ethics Statement

All animal experiments were approved by the Institutional Animal Care and Use Ethics Committee of Shandong Agricultural University (No.2004005, Figure S1) and performed in accordance with the “Guidelines for Experimental Animals” of the Ministry of Science and Technology (Beijing, China). All surgery was performed according to Recommendations proposed by European Commission (1997), and all efforts were made to minimize suffering.

### Animal Sample Collection and RNA Extraction

In this study, five healthy Laoshan dairy goats in the third lactation period (four years old) were used. The mammary gland tissues were collected respectively at peak (90 days postpartum) and late lactation (210 days postpartum) using surgery and immediately frozen in liquid nitrogen. Total RNAs for five tissues in the same lactation period were respectively extracted using TRIzol reagent (Invitrogen, Carlsbad, CA, USA) according to the manufacturer’s instructions, subsequently be homogenized and pooled for library construction and Solexa sequencing. The quantity and integrity of the total RNA was assessed using an Agilent 2100 Bioanalyzer (Agilent Technologies, USA). Total RNA was stored at −80*°*C until further use.

### Small RNA Library Construction and Solexa Sequencing

The small RNA (sRNA) libraries were constructed using the homogenized and pooled total RNAs of five individuals from peak and late lactation respectively. The overall flow of sRNA library construction, Solexa sequencing and bioinformatics analysis was shown schematically in Figure S2 and Figure S3, respectively. From each sample, 20 µg of total RNA was used for the library construction using a Small RNA Sample Prep Kit (Illumina, USA) following the manufacturer’s instructions with minor modifications. Briefly, the 18∼30 nt fraction of total RNA was excised and purified following 15% Tris-Borate-EDTA (TBE) denaturing polyacrylamide gel electrophoresis (PAGE). Subsequently, 3′ and 5′ RNA adaptors were respectively ligated using T4 RNA ligase. The adaptor-ligated small RNAs were subjected to RT-PCR amplification, and the cDNA was further amplified with 15 PCR cycles. The PCR products (90 bp, small RNA + adaptors) were purified on 4% agarose gels and used for sequencing analysis on a Illumina 1G Genome Analyzer (Illumina, San Diego, CA, USA) at the Beijing Genomics Institute (BGI), Shenzhen, P.R. China. Then, the image files were generated using a Solexa sequencer and processed to produce digital-quality data. After masking the adaptor sequences and removing contamination, the clean reads were processed for computational analysis. The sequenced short reads data have been deposited to the GEO in NCBI with accession number: No. GSE39484.

### Bioinformatics Analysis for Small RNAs

The basic figure from sequencing was converted into sequence data (raw data or raw reads) using the base-calling step. The raw data were processed to obtain clean reads through the elimination of the following aspects: a) low quality reads; b) reads without 3′ primer; c) reads with 5′ primer contaminants; d) reads without the inserted tag; e) reads with poly(A); and f) reads shorter than 18 nt. The clean reads were mapped to the *Ovis aries* genome (UCSC, 2010, ISGC *Ovis aries* 1.0/oviAri1 [18]) using SOAPv1.11 software [19] to analyze their expression and distribution. Sequences with perfect matches were retained for further analysis. The sequences were aligned against the known miRNAs precursors and mature miRNAs deposited in the miRBase 18.0 to identify conserved miRNAs. The clean reads were compared against the small RNAs (rRNAs, tRNAs, snRNAs, snoRNA, miRNA) deposited in the GenBank and Rfam (http://www.sanger.ac.uk/resources/databases/rfam.html) databases to annotate the small RNA sequences using tag2annotation software (developed by BGI). Because some small RNA tags might be mapped to more than one category, we used the following priority rules to ensure that every unique small RNA was mapped to only one annotation: rRNA etc. (GenBank > Rfam) > known miRNA > repeat > exon > intron. The unannotated sequences were used to predict potential novel miRNA candidates.

To further analyze the RNA secondary structures of potential miRNA candidates, we used the Mfold3.2 software [20] to predict the RNA secondary structures, and subjected each sequence to further analysis using the MIREAPv0.2 software (https://sourceforge.net/projects/mireap/) [21] under the following parameter settings according to Zuker and Jacobson [22]: a) miRNA sequence length (18∼26 nt); b) miRNA reference sequence length (20∼24 nt); c) Minimal depth of Drosha/Dicer cutting site (3); d) Maximal copy number of miRNAs on reference (20); e) Maximal free energy allowed for a miRNA precursor (−18 kcal/mol); f) Maximal space between miRNA and miRNA* (35); g) Minimal base pairs of miRNA and miRNA* (14); h) Maximal bulge of miRNA and miRNA* (4); i) Maximal asymmetry of miRNA/miRNA* duplex (5); and j) Length of the sequence flanking the miRNA precursor (10). Stem-loop hairpins were considered typically in accordance with the following three criteria [23]: mature miRNAs are present in one arm of the hairpin precursors, which lack large internal loops or bulges; the secondary structures of the hairpins are steady, with a free energy of hybridization lower than −18 kcal/mol; and hairpins are located in intergenic regions or introns. Genes with sequences and structures that fulfilled the three criteria, forming perfect stem-loop structures, were considered as potential miRNA candidates. Finally, all remaining novel miRNA candidates were subjected to MiPred (http://www.bioinf.seu.edu.cn/miRNA/) to filter out pseudo-pre-miRNAs using the following settings: Minimum free energy >−20 kcal/mol or P-value >0.05 [24].

### Target Genes Prediction of Differential Expression microRNAs

Because no 3′-UTR database is currently available, the putative target genes for differentially expressed miRNAs was performed by aligning the miRNA sequences with the goat EST database in NCBI (http://www.ncbi.nlm.nih.gov/sites/entrez?db=nucest&cmd=Search&dopt=DocSum&term=txid9925%5BOrganism%3Anoexp%5D), according to the following rules as guidelines suggested by Allen [25] and Schwab [26]: a) No more than four mismatches between miRNA and target genes (G-U bases count as 0.5 mismatches); b) No more than two adjacent mismatches in the miRNA/target duplex; c) No adjacent mismatches in positions 2∼12 of the miRNA/target duplex (5′ of the miRNA); d) No mismatches in positions 10∼11 of the miRNA/target duplex; e) No more than 2.5 mismatches in positions 1∼12 of the miRNA/target duplex (5′ of the miRNA); and f) The minimum free energy (MFE) of the miRNA/target duplex should be ≥75% of the MFE of the miRNA bound to its perfect complement. More strictly, at most two mismatches between the miRNA sequences and the potential miRNA targets were allowed in this study.

### GO Annotation and KEGG Pathway Analysis of Target Genes

To comprehensively describe the properties of genes and gene products, InterProScan (http://www.geneontology.org/GO.annotation.interproscan.shtml [27]) and Blast2go (http://www.blast2go.com/b2ghome [28]) were used to execute GO annotation and enrichment analysis from three ontologies: molecular function, cellular component and biological process. The GO terms were significantly enriched in the predicted target gene candidates of the miRNAs compared with the reference gene background and the genes corresponding to certain biological functions. This method maps all target gene candidates to GO terms in the database (http://geneontology.org/) [29], calculates the gene numbers for each term, and applies a hypergeometric test to find significantly enriched GO terms in the target gene candidates compared with the reference gene background. A Bonferroni correction was applied to obtain a corrected *P*-value. The GO terms with corrected P-values ≤0.5 are defined as significantly enriched in the target gene candidates, using the following calculation:
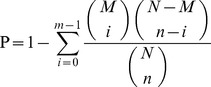



In the formula above, N is the number of all genes with GO annotations; n is the number of target gene candidates in N; M is the number of all genes that are annotated to a certain GO term; and m is the number of target gene candidates in M.

To obtain the significantly enriched metabolic or signal transduction pathways in the target gene candidates compared with the whole reference gene background, Cytoscape software V2.8.2 (http://www.cytoscape.org/) [30] and the ClueGO plug-in (http://apps.cytoscape.org/apps/cluego) [31] were used to decipher the KEGG (Kyoto Encyclopedia of Genes and Genomes, http://www.genome.jp/kegg/) [32] pathway and understand their biological functions. The genes with FDR ≤0.5 were considered as significantly enriched in target gene candidates. The formula was the same as that used in the GO analysis. However, N is the number of all genes with KEGG annotations, n is the number of target gene candidates in N, M is the number of all genes annotated to a certain pathway, and m is the number of target gene candidates in M.

### Differential Expression Analysis and Hierarchical Clustering of microRNAs

To compare the differential miRNA expression between peak and late lactation, the expression abundances of miRNAs in the two samples were normalized to obtain the expression of transcripts per million. If the normalized expression (NE) value of a given miRNA is zero, the expression value was modified to 0.01. If the normalized expression of a given miRNA is less than 1 in both libraries, it was removed in future differential expression analyses. The fold-change and *P*-value were calculated from the normalized expression using the following formulas:

Normalized expression  =  (Actual miRNA sequencing reads count/Total clean reads count) ×1,000,000.

Fold change  =  Log2 (H/S).

*P*-value:
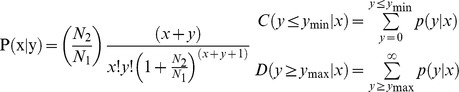



The N_1_ and x represent the total count of clean reads and normalized expression, respectively, for a given miRNA in the peak lactation sRNA library. The N_2_ and y represent the total count of clean reads and normalized expression respectively, for a given miRNA in the late lactation sRNA library.

The hierarchical clustering of miRNA expression was performed using PermutMatrix software with Pearson distance [33]. The relative expression frequency for each miRNA was calculated as the number of sequences for each miRNA in a library divided by the total of number of sequence reads for that library.

### Real-time Quantitative RT-PCR

Differentially expressed miRNAs were validated using relative real-time quantitative RT-PCR according to the manufacturer’s protocol. Real-time quantitative PCR was performed using the Mx3000p™ SYBR® Green real-time quantitative PCR Analyzer (Stratagene, USA). Briefly, 2 µg of miRNA was reverse transcribed using the One Step PrimeScript® miRNA cDNA Synthesis Kit (TaKaRa Biotechnology Co., Ltd., Japan, D350A). The reverse transcription reaction system included 10 µL of 2X miRNA Reaction Buffer, 2 µL of 0.1% BSA, 2 µL of miRNA PrimeScript® RT Enzyme Mix, 2 µL of total RNA (10 pg/µL∼1 µg/µL), and RNase-Free dH_2_O to a final volume of 20 µL. The RT-PCR program was set to 37*°*C for 60 min followed by 85*°*C for 5 sec. The cDNA products were stored at −20*°*C. The relative real-time quantitative PCR was performed with SYBR® Premix Ex Taq™ ? (TaKaRa Biotechnology Co., Ltd., Japan, DRR081A). The reaction solution was prepared on ice, and comprised 10 µL of 2X SYBR® Premix Ex Taq™ ?, 0.8 µL of PCR Forward Primer (10 µM), 0.8 µL of Uni-miR qPCR Primer (10 µM), 0.4 µL of 50X ROX Reference Dye ?, 2 µL of cDNA, and dH_2_O to a final volume of 20 µL. The reaction mixtures were incubated in a 96-well plate at 95*°*C for 30 sec, followed by 40 cycles of 95*°*C for 5 sec, 60*°*C for 30 sec and 72*°*C for 30 sec. All reactions were performed in triplicate. The primers for the miRNAs had the same sequences as the *Bos taurus* miRNAs with appropriate adjustments at their 5′ terminus. Mx3000/Mx Pro software (Stratagene, USA) was used to construct a melting curve. Standard curves with 5 fold dilutions (made from the pool consisting of equal amounts of the 96 cDNA samples) were performed for each assay, and the PCR efficiency calculations were based on the slopes of the standard curves. The absolute amount of each miRNA was calculated using the 2^−△△CT^ method [34] according to the standard curve. The housekeeping gene U6 was used as an endogenous control. Each sample was replicated for three times. The miRNA level in mammary gland samples was determined individually. The each miRNA level was expressed as 2^−△△CT^mean ± SE. One-way ANOVA was used to examine the significance of differential expression level in each mature/novel miRNA between peak and late lactation, and the difference was considered as significant when *P*<0.05.

## Results

### Small RNAs Library Construction and Solexa Sequencing

To identify miRNAs involved in mammary gland physiology and lactation, two small RNAs libraries pooled from peak and late lactation mammary gland tissues of five individuals were constructed respectively, and sequenced using an Illumina/Solexa 1G high-throughput sequencer (Figure S2 and Figure S3). As a result, a total of 19,834,279 and 10,083,672 raw reads were provided respectively, for peak and late lactation libraries. After removing the low quality reads, adaptors, and insufficient tags, 19,044,002 and 7,385,833 clean reads of 18∼30 nt were ultimately obtained (Table 1). Of these, 8,941,279 peak lactation sequences and 3,916,179 late lactation sequences, which account for 46.95% and 53.02% of total reads, respectively (Table 1), were perfectly mapped to the *Ovis aries* genome (UCSC, 2010, ISGC *Ovis aries* 1.0/oviAri1 [18]). All identical sequence reads were grouped together to simplify the sequencing data, a total of 466,727 and 259,250 unique sequences for peak lactation and late lactation were remained respectively for further analysis (Table 1).

**Figure 1:**
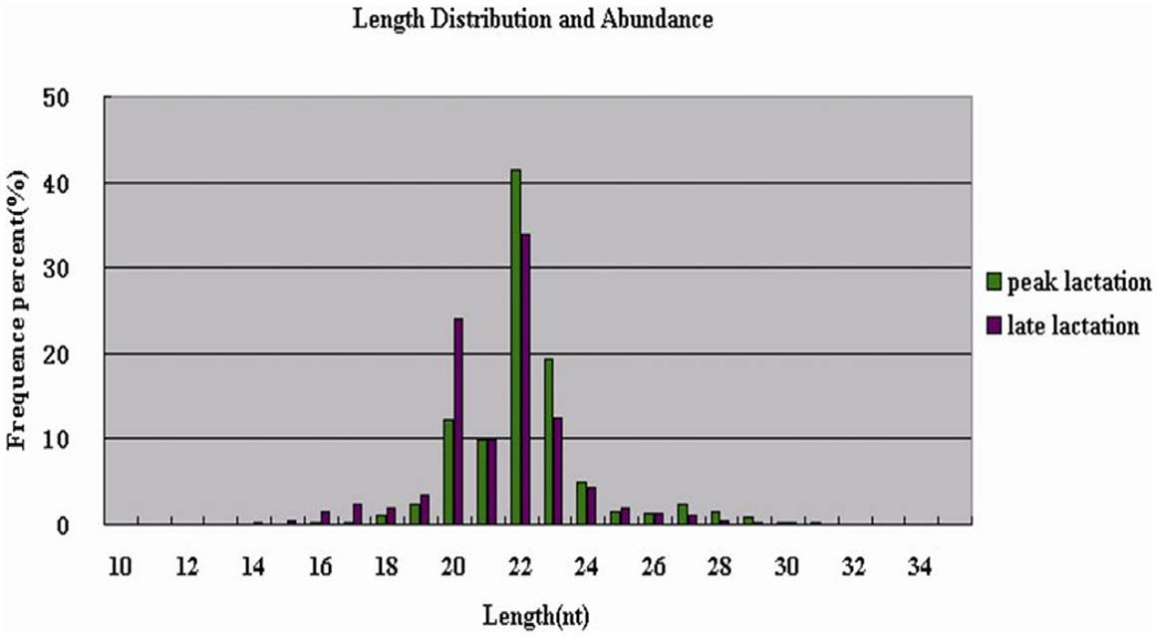
Length distribution and abundance of sequences in peak and late lactation. Sequence length distribution of clean reads based on the abundance and distinct sequences; the most abundant size class was 22 nt, followed by 20 nt, 23 nt and 21 nt.

**Figure 2:**
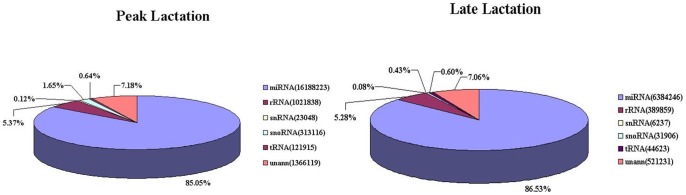
Distribution of small RNAs among different categories in peak and late lactation. The clean reads were annotated and classified as miRNA, rRNA, tRNA, snRNA and snoRNA in GenBank and Rfam databases, and partial reads were not annotated.

Of the total small sequences in the two libraries, the size distribution of the small RNAs was similar (Figure 1). The lengths of the majority of small RNAs were 19∼24 nt, and the most abundant size class in the small RNA sequences distribution was 22 nt, which accounted for 41.48% and 33.96% in peak and late lactation respectively, followed by 20 nt (12.31%, 23.96%), 23 nt (19.32%, 12.53%) and 21 nt (9.91%, 9.96%), which are typical of small RNA Dicer-processed products and are consistent with the known 18∼25 nt range for miRNAs. To assess the efficiency of high-throughput sequencing for sRNA detection, all sequence reads were annotated and classified through alignment with GenBank and Rfam databases. The classification annotation revealed (Figure 2) 19,034,259 and 7,378,102 reads were annotated and classified as miRNA, rRNA, tRNA, snRNA and snoRNA, 1,366,119 (309,527 unique sRNAs, accounted for 7.17% of total reads) and 521,231 (169,990 unique sRNAs, accounted for 7.06% of total reads) reads were unannotated in peak and late lactation, and remained for further analysis of novel miRNA candidates.

**Table 1:** Summary of Solexa sequencing data for small RNAs in peak and late lactation.

Categories	Peak Lactation		Late Lactation	
	Unique sRNAs	Total sRNAs	Unique sRNAs	Total sRNAs
Raw reads number	–	19,834,279	–	10,083,672
Clead reads	466,727	19,044,002	259,250	7,385,833
Perfect match to Ovis aries Genome	69,244	8,941,279	32,509	3,916,179
Specific sequences	399,868	667,325	192,391	299,916
Common	Unique sRNAs: 66,859		Total sRNAs: 25,462,594	
Total	Unique sRNAs: 659,118		Total sRNAs: 26,429,835	

### Known Conserved microRNAs and Differential Expression in Goat Mammary Gland

**Figure 3:**
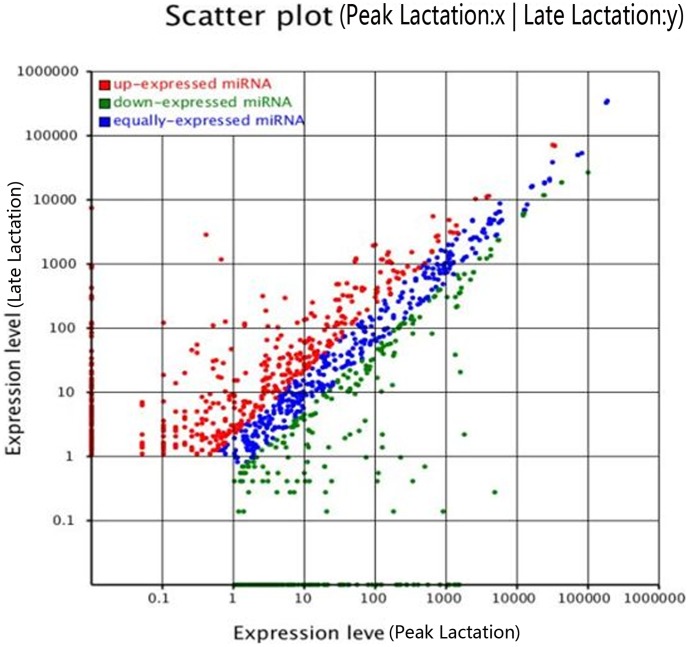
Comparison of expression levels of miRNAs in peak and late lactation. The X- and Y-axes show the expression levels of miRNAs in the two samples. The red points represent miRNAs with ratios >2; the blue points represent miRNAs with 1/2<ratios<2; the green points represent miRNAs with ratios <1/2. Ratios  = miRNA expression levels of late lactation/miRNAs expression levels of peak lactation.

To identify the known miRNAs in our sequenced set of sRNAs, we compared the sequences recovered from our libraries with the repository of mature animal-miRNAs in miRBase 18.0 using MIREAPv0.2 software. After blastn searches (number mismatch ≤3) and further sequences analysis, 15,687,114 and 6,134,001 sequences (representing 46,610 and 35,221 unique sRNAs, respectively) in peak lactation and late lactation had perfect matches to known animal miRNAs deposited in miRBase 18.0, a total of 934 known miRNAs were co-expressed, 104 miRNAs were peak lactation-specific and 75 were late lactation-specific (Table S1). Compared with miRNA expression in peak lactation, 362 miRNAs in late lactation were significantly up-regulated with P≤0.01, while 272 miRNAs were significantly down-regulated with P≤0.01 (Figure 3 and Table S1). We analyzed the number of reads for conserved miRNA and found it has large divergence in the expression frequency among these miRNAs. In peak lactation, miR-143, miR-143-3p and miR-148a-3p were predominately expressed with more than 100,000 reads, and these miRNAs constituted 30.71% of the total sequencing reads, suggesting they are abundantly expressed during this period. The sequencing frequencies of 628 miRNAs were much lower than the 10 reads (Table S1). However, in late lactation, miR-143, let-7, miR-21, miR-148, miR-30, miR-146, miR-107 and miR-103 were the most abundant, each with more than 100,000 reads. A total of 327 miRNAs displayed the lowest sequencing frequencies, with no more than 10 reads in peak or late lactation. In two libraries, let-7, miR-143, miR-148, miR-378, miR-146 and miR-21 were detected with high abundance (Table S1). The 10 most abundance co-expressed miRNAs during peak and late lactation were listed in Table 2.

**Table 2:** The 10 most abundance co-expressed miRNAs during peak and late lactation.

miRNAs	Reads		Nor_ Reads		F_ change	*P*_ Value	Sig_ level
	Peak lactation	Late lactation	Peak lactation	Late lactation			
miR-143	3,663,628	2,501,394	192,377	338,674	0.82	0	#
miR-143-3p	3,474,077	2,361,222	182,423	319,696	0.81	0	#
miR-148a-3p	1,948,637	193,553	102,322	26,205	−1.97	0	*
let-7-5p	1,569,878	386,744	82,434	52,362	−0.65	0	#
let-7b	1,414,825	368,460	74,292	49,887	−0.57	0	#
let-7b-5p	1,374,801	361,024	72,190	48,880	−0.56	0	#
miR-30a	818,766	135,764	42,993	18,381	−1.23	0	*
miR-30a-5p	818,248	134,361	42,966	18,191	−1.24	0	*
miR-378e	654,953	511,109	34,392	69,201	1.01	0	*
miR-378d	654,554	510,815	34,371	69,161	1.01	0	*

**Note:** Nor_Reads: Normalized reads, the results of Solexa sequencing, Normalization formula: Normalized expression = Actual miRNA count/Total count of clean reads×1,000,000; F_change: Fold_changes (Log2 Late lactation/Peak lactation), fold changes of miRNAs in both samples, – represents down regulation in late lactation; *P*_Value: *P* values manifest the significance of miRNAs differential expression between two samples; Sig_level: Significance_level, # represents no significant difference, * represents significant difference;

The main purpose of the present study was to identify miRNAs involved in mammary gland development. According to the changes in relative miRNA abundance between the two libraries, 697 miRNAs were significantly differently expressed between peak and late lactation (Table S1). As shown in Table 3 and Table S1, the majority of differentially expressed miRNAs in fold-change ranges from 1 to 15 fold, only 9 miRNAs showed differences of greater than 15-fold between the two libraries. Among the up-regulated miRNAs, miR-17a* has the highest fold-change of at least 19 fold, while in the down-regulated miRNAs, miR-881 has the highest fold-change of at least 17 fold, and followed by miR-3175, miR-3966 with the fold changes of at least 17 fold (Table 3 and Table S1). The hierarchical clustering of the two tissues based on the relative expression frequencies of the miRNAs also suggested that miRNA expression was different in peak and late lactation (Figure S4), and all differentially expressed miRNAs were clustered in one by six cluster.

**Table 3:** The 10 most different fold changes miRNAs between peak and late lactation.

miRNAs	Reads		Nor_ Reads		F_ change	*P*_ Value	Sig_ level
	Peak lactation	Late lactation	Peak lactation	Late lactation			
miR-17a*	0	54,914	0.01	7,435	19.50	0	**
miR-5425	0	6,766	0.01	916	16.48	0	**
miR-4754	0	6,326	0.01	856	16.37	0	**
miR-3138	10,637	0	559	0.01	−15.77	0	**
miR-125a*	15,723	0	826	0.01	−16.33	0	**
miR-125*	20,053	0	1,052	0.01	−16.68	0	**
miR-29-3p	21,450	0	1,126	0.01	−16.78	0	**
miR-3966	25,136	0	1,320	0.01	−17.01	0	**
miR-3175	27,377	0	1,438	0.01	−17.13	0	**
miR-881	29,370	0	1,542	0.01	−17.23	0	**

**Note:** Nor_Reads: Normalized reads, the results of Solexa sequencing, Normalization formula: Normalized expression = Actual miRNA count/Total count of clean reads×1,000,000; F_change: Fold_changes (Log2 Late lactation/Peak lactation), fold changes of miRNAs in both samples, – represents down regulation in late lactation; *P*_Value: *P* values manifest the significance of miRNAs differential expression between two samples; Sig_level: Significance_level, # represents no significant difference, * represents significant difference;

### Identification of Novel microRNAs Candidates

In additional to profiling known miRNAs, Solexa high-throughput sequencing has a special advantage for discovering functionally important novel miRNAs that might not be detected using traditional methods. In this study, 1,366,119 and 521,231 unannotated small RNAs, separately presenting 309,527 and 169,990 unique sRNAs matching to the *Ovis aries* genome (UCSC, 2010, ISGC *Ovis aries* 1.0/oviAri1) (Figure 2), were used to predict potential novel miRNA candidates. To determine whether these small RNA sequences were genuine goat miRNA, we explored their hairpin structures, Dicer cleavage sites and minimal free energies using MIREAPv0.2 software (https://sourceforge.net/projects/mireap/) [21]. Mfold [20] and MiPred [24] softwares were also used to predict the typical secondary structures of the miRNA precursors and remove pseudo-pre-miRNAs. In total, 31 potential novel miRNA candidates with lengths ranging from 20 to 24 nt and reads ranging from 5 to 708 were obtained from peak and late lactation libraries, which possessed a typical stem-loop structure and free energy ranging from −19.6 Kcal/mol to −66.7 Kcal/mol (Table S2).

### Validation of microRNAs Expression with Quantitative RT-PCR

**Figure 4:**
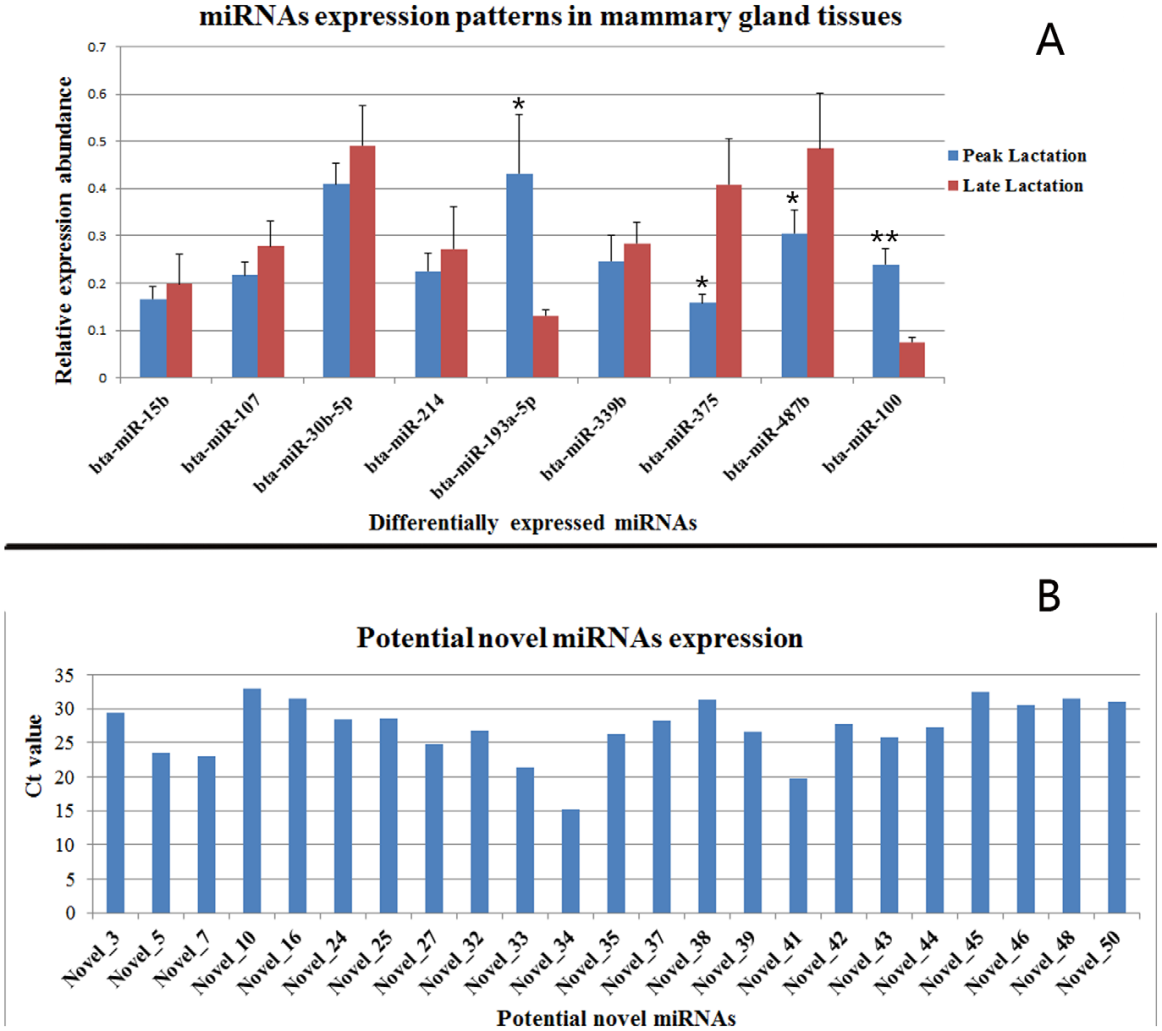
qRT-PCR validation of the identified miRNAs using Solexa sequencing technology. **A:** Real-time RT-PCR results for 9 known miRNAs in peak and late lactation. The relative quantification of expression was calculated using the 2^−△△CT^ method after the threshold cycle (Ct) and was normalized with the Ct of U6. The relative expression levels were presented as the 2^−△△Ct^ means ± SE. The error bars indicate the standard error of the 2^−△△Ct^ mean values. * represents *p*<0.05, ** represents *p*<0.01. **B:** Potential novel miRNAs expression in mammary gland. Total RNA pooled from five individuals of two lactation periods were used for qRT-PCR, the relative expression abundance were expressed as Ct value, each sample was replicated for three times.

To validate the reliability of the sequencing data, we applied relative real-time quantitative RT-PCR to compare the expression levels of the differentially expressed miRNAs and newly identified miRNAs. The five individuals in each lactation period (peak and late lactation) were subjected respectively to qRT-PCR, the expression levels of 9 differentially expressed miRNAs were selected randomly and validated in peak and late lactation. The results are shown in Figure 4A, bta-miR-15b, bta-miR-107, bta-miR-30b-5p, bta-miR-214, bta-miR-193a-5p, bta-miR-339b, bta-miR-375, bta-miR-487b, and bta-miR-100 were differentially expressed in peak and late lactation, and the expression levels of bta-miR-15b, bta-miR-107, bta-miR-30b-5p, bta-miR-214, bta-miR-339b, bta-miR-375, and bta-miR-487b in late lactation tissue were higher than the expression levels in peak lactation, bta-miR-100 was down regulated in late lactation compared with peak lactation, the expression pattern was consistent with the Solexa sequencing results (Table S1), only bta-miR-107 was not consist with Solexa sequencing results, this may be caused by deviation of qRT-PCR. Furthermore, we also verified the expression of 31 novel miRNAs using qRT-PCR, in which 23 were validated, 8 were not detected (Figure 4B). The primers for the validated miRNAs are shown in Table 4.

**Table 4:** Summary of miRNA primers used in real-time RT-PCR.

Primer	Sequences (5′→3′)	Length (nt)	GC (%)	Tm
U6	CAAGGATGACACGCAAATTCG	21	47.6	69
bta-miR-15b	CGTAGCAGCACATCATGGTTTACA	24	45.8	72
bta-miR-107	GAGCAGCATTGTACAGGGCTATC	23	52.2	73
bta-miR-30b-5p	GCCGAAACATCCTACACTCAGCT	23	52.2	73
bta-miR-214	AGCAGGCACAGACAGGCAGT	20	60.0	72
bta-miR-193a-5p	GTCTTTGCGGGCGAGATGA	19	57.9	69
bta-miR-339b	CCATATCTGTCCTCCAGGAGCTC	23	56.5	75
bta-miR-375	TTCGTTCGGCTCGCGTGA	18	61.1	69
bta-miR-487b	GCAATCGTACAGGGTCATCCAC	22	54.5	70
bta-miR-339b	CCATATCTGTCCTCCAGGAGCTC	23	56.5	75
bta-miR-100	AACCCGTAGATCCGAACTTGTG	22	50.0	71
Novel_3	TACGAGGACCTCCAGAACGACC	22	59.1	65
Novel_5	CCAGGCTAGGAGAAATGATTGG	22	50.0	62
Novel_7	CCCAAACCAGTTGTGCCTGTAG	22	54.6	64
Novel_10	AGCGCAAATGAACGTTTTGG	20	45.0	63
Novel_16	CCAGGGATGTAGCTCCTAGTGC	22	59.1	63
Novel_24	TGGGAGTCGCTGAGAGGATGGA	22	60.9	69
Novel_25	CAGGGAGAAGGTGAGCAGAGGA	22	59.1	65
Novel_27	TGTGAGAGAAGAGGACGCCTGG	22	59.1	66
Novel_32	ATATATCCAGGGGACGCCGTT	21	52.4	64
Novel_33	CCGCCGGTAAAAAATTGATTTGACT	25	40.0	67
Novel_34	GCGTCAAGTGATGGAGAAGATGA	23	47.8	64
Novel_35	AATTCCGGTCGCTGTGCTCTCG	22	59.1	70
Novel_37	GCGAACCTGAGCAAACTTTTT	21	42.9	61
Novel_38	CACTGATTGGGGTAGATGTGAGA	23	47.8	62
Novel_39	CAGCAACTAAAGATCCCTCAGG	22	50.0	60
Novel_41	ATAGAGAGGGATGGGCTGGG	20	60.0	63
Novel_42	CGCTGAACAAACTTTTTGGCCA	22	45.5	66
Novel_43	TGCCAAGCCCACGTTCAAAGG	21	57.1	70
Novel_44	TGGGGAAGCGCAGGAAACAGT	21	57.1	68
Novel_45	TGAGTTAAAGGAAAAGGAAAGT	22	31.8	54
Novel_46	AATGTACTTGTGGAGTTGGAG	21	42.9	54
Novel_48	TGGACAACTGAAGCTAGCACAG	22	50.0	61
Novel_50	ATAGAGCCCCGGTAGACTGT	20	55.0	58

### MicroRNA Target Genes Prediction, GO Enrichment and KEGG Pathway Analysis

To further understand the physiological functions and biology processes involved these miRNAs during mammary gland development and lactation, target gene prediction was performed based on miRNA/mRNA interactions to provide some molecular insight into the processes. The results were analyzed using MIREAPv0.2 software (MATERIALS & METHODS). A total of 762,557 annotated mRNA transcripts were predicted as putative target genes for 712 differentially expressed miRNAs (Table S3).

**Figure 5:**
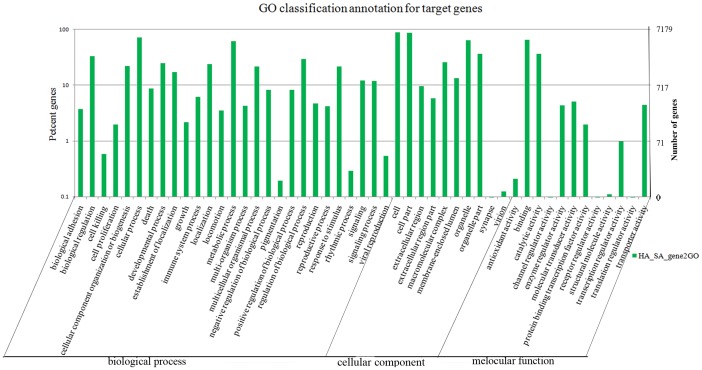
Part gene ontology classification annotated by gene2go for predicted target genes. The figure shows partial GO enrichment for the predicted target genes in molecular function, cellular component and biological processes.

The predicted target genes were classified according to KEGG function annotations to identify the pathways that were actively regulated by miRNAs in mammary gland tissue (Table S4). The KEGG Pathway annotation showed 17,196 target genes that were annotated for 261 biological processes with P<1, and most of target genes were involved in cellular metabolism, disease and signal transduction. The most enriched pathway annotated was metabolic pathways, with 1,251 annotated genes representing 7.27% followed by Huntington’s disease pathways (3.43%), Parkinson’s disease pathways (3.31%), and oxidative phosphorylation (3.25%). GO is an international standardized classification system for gene annotations that provides insight into the molecular functions of genes in various biological processes [29]. The GO enrichment analysis from cellular components showed that 25,295 genes were termed from component ontology with a P-value ≤1. Moreover, 51% of the genes were clustered into intracellular regions. The analysis of molecular function showed that 8,879 genes were assigned different functions, and most of the functions were related to binding activity, with 4,834 (occupied 54.44%) annotated genes. The analysis of biological processes showed that 34,847 genes were involved in cellular or metabolic processes, with 16.12% and 13.80%, respectively (Table S5). Partial GO annotations for predicted target genes are shown in Figure 5.

## Discussion

MicroRNAs, as key components of most of the regulatory events, play important roles at the post-transcriptional level in various developmental and physiological processes [7], [8]. Although most conserved and high-abundant miRNAs had been identified by traditional cloning method or bioinformatics prediction [35], [36], following the development of sequencing techniques and bioinformatics, a large number of miRNAs have been found in various species. Illumina/Solexa high-throughput sequencing is a small RNA digitalization analysis platform based on sequencing-by-synthesis (SBS) technique, which has special advantages for small RNAs sequencing because of its high-throughput, high-accuracy, high-repeatability and low signal-to-noise ratio [37]. Currently, Solexa high-throughput sequencing is used as a powerful tool for the identification of miRNAs and was widely used for small RNAs digital expression profiling in various organisms [38], [39], [40], [41]. The results are typically validated using real-time quantitative RT-PCR, northern blot and microarray analysis [42], [43], [44]. In this study, the amount of data obtained using Solexa sequencing covered almost all small RNA. The length distribution showed that more than 89% of the small RNA sequences were primarily distributed in 19∼24 nt in the two libraries, which is consistent with the typical size of mature mammalian miRNA from Dicer digestion products [45], these results were consistent with those from previous studies [15], [39], [46]. The size distribution of the small RNAs from peak and late lactation was similar, while there were also differences in the length distribution and abundance in the two stages (Figure 1). These results shown that miRNAs with different length might perform different functions during different developmental stages or different physiological periods. The Solexa sequencing reads were aligned against the *Ovis aries* genome (UCSC, ISGC *Ovis aries* 1.0/oviAri1) (Table 1), and annotated in GenBank and Rfam databases (Figure 2), the results indicate that the high-throughput sequencing data were highly enriched for small RNAs sequences.

Analyzing the spatiotemporal expression patterns of miRNAs would useful information for their physiological functions [47]. In this study, miRNAs comprised a large proportion of the goat small RNA libraries (16,188,223 and 6,384,246 sequences in peak and late lactation, respectively. Figure 2), indicating that miRNAs are primarily involved in the regulation of gene expression in the lactating mammary gland. One striking observation was that many miRNAs exhibited significantly differential expression patterns between peak lactation and late lactation (Figure 3, Figure 4, Table 3 and Table S1). This result led to the general conclusion that miRNAs are highly stage-specific regulatory elements. It could be considered that miRNAs which were highly expressed in peak lactation might be involved in the specific physiological processes, such as high milk yield, milk ingredients. The analysis of conserved and differentially expressed miRNAs shown that the most abundant miRNA was miR-143, which was represented approximately 3,000,000 sequence reads in peak and late lactation. It was reported that miR-143 plays an important role in many pathological tissues as a tumor suppressor by inhibiting cell apoptosis or promoting cell proliferation [48], [49]. The predominance of miR-143 might be consistent with its well-established function in lactating mammary gland physiology. Thus, miR-143 could be involved not only in breast diseases, but also mammary development, and might be associated with mammary gland lactation or milk ingredients. Three other miRNAs, miR-148a-3p, let-7-5p and let-7b were also identified as high-count sequences with more than 1,000,000 reads in both libraries (Table 2 and Tabel S1). It has been reported that the sequential stage-specific expression of the let-7 regulates developmental timing in *Caenorhahditis elegans* [50]. In recent years, numerous studies have indicated that miR-148a is involved in cell differentiation or proliferation. Zhang et al. [51] reported that miR-148a targets the ROCK1 gene to promote myogenic differentiation. Liffers et al. [52] reported that miR-148a is down-regulated in human pancreatic ductal adenocarcinomas and regulates cell survival through targeting CDC25B. In our study, miR-148a has high abundance, it may be involve in mammary gland cell proliferation and apoptosis and play an important role in mammary gland physiology or lactation. Compared with the above four miRNAs, 393 miRNA were expressed at low levels, with less than 100 reads in the two mammary libraries. There were 104 miRNAs that existed solely in peak lactation, and 75 miRNAs were present in late lactation (Table S1). These variations in abundance could also reflect differences in the roles of these miRNAs in terms of the regulation of mammary physiology and lactation [53].

In additional to profiling known miRNAs, high-throughput sequencing has a special advantage for discovering functionally important novel miRNAs that might not be detected using traditional methods. Currently, many computational programs, such as miRSan, miRseeker and findMiRNA, have been developed for predicting novel miRNA [54], and all of these programs were designed based on specific miRNA hairpin stem-loop secondary structures and high negative minimal folding free energies, it is extremely challenging to define novel miRNAs because other RNAs, particularly mRNA, also have similar stem-loop structures and high negative minimal folding free energies [55]. Previously, miRNAs were defined as noncoding small RNAs that fulfill a combination of expression and biogenesis criteria [56], [57]. First, a mature miRNA should be expressed as a distinct transcript of approximately 22 nucleotides that is detectable using qRT-PCR or other experimental means. Second, a mature miRNA should originate from a precursor with a characteristic secondary structure, such as a hairpin or fold-back that does not contain large internal loops or bulges. Finally, the mature miRNA should occupy the stem region of the hairpin structure. Although hairpin structures are common in eukaryotic genomes, these structures are not a unique feature of miRNAs. Many random inverted repeats (termed pseudo-hairpins) can also fold into dysfunctional hairpins [58]. In this study, MIREAP (https://sourceforge.net/projects/mireap/) were used to predict the conserved and novel miRNAs by exploring the secondary structure, Dicer cleavage site and minimum free energy [21]. Subsequently, we eliminated the reads with abundances of less than two, and used the Mfold and MiPred softwares to further analyze their typical stem-loop structures and filter out the pseudo-novel miRNAs. The novel miRNAs were further validated using real-time quantitative PCR (Figure 4B). Of the 31 potential novel miRNA candidates, 23 were validated, 8 novel miRNAs were not obtained by qRT-PCR, it may be the inappropriate primers design, or the very low expression abundance, or the false positive of novel miRNAs, which need further experimental verifications.

MiRNAs function by incomplete complementary binding the 3′ UTR of target mRNAs to regulate protein expression or degrade target mRNA [5], [6]. So far, although several bioinformatics tools have been developed, such as miRanda, RNAhybrid, PicTar, none of the available computational methods can predict miRNA targets accurately and they all give results with higher false positive rates [59], [60], because the detailed mechanism of interaction between miRNA and its target transcripts is not clear. Moreover, there is no 3′ UTR database available, it is more difficult to predict targets of goat miRNAs. Alternatively, to further provide the insight into the physiological functions of miRNAs involving in lactating mammary gland, the presumable target genes for differentially expressed miRNAs were predicted by aligning miRNA sequences to goat ESTs following the rules of target prediction suggested by Allen [25] and Schwab [26] (Materials and Methods) using MIREAP and TargetScan 6.0 software to maximally eliminate some false positives. GO annotation and KEGG Pathway analyses could also obtain a better understanding from the molecular functions, cellular components, and biological processes of target genes. The analysis of GO and KEGG shown that the putative target genes appear to be involved in a wide variety of biological processes. Ranging from genes encoding transcription factors involved in transcription regulation to genes encoding enzymes involved in metabolism, genes regulating transport, genes encoding various kinases, genes regulating oxidative reduction and genes encoding isomarase and helicase (Table S3, Table S4 and Table S5). The GO enrichment analysis revealed that more than 51% of the genes were annotated to intracellular component ontology, more than 16% of the genes were involved in cellular processes and approximately 54% of the genes had binding functions (Table S5). KEGG analysis showed that approximately 7% of the genes were committed to a metabolic pathway (Table S4). These results indicated that some miRNAs might be involved in mammary gland lactation and physiology, and function in mammary gland cell proliferation, apoptosis, and differentiation. Although a large number of target gene candidates were predicted using bioinformatics tools, validation of the relationship between miRNAs and mRNA transcripts need further more biological experimental evidences.

### Conclusions

Solexa high-throughput sequencing provided a strong platform for the study of small RNAs in dairy goat mammary gland physiology and lactation. In this study, we constructed two small RNA libraries from mammary gland tissues in peak and late lactation, and obtained high-quality small RNA expression profiles, which lead to the significant enrichment of a *Capra hircus* miRNA dataset. The present study is the first to examine the goat mammary gland miRNA expression profile and evaluate miRNA function during mammary gland lactation through the identification of differentially expressed miRNAs between peak and late lactation. The identification of 23 novel miRNAs highlights the important function of low abundant and less conserved miRNAs in the physiology of specific tissues. Target gene predictions for 697 significantly differentially expressed miRNAs, functional annotations and pathway analyses in GO and KEGG databases demonstrated the regulation of genes targeted by miRNAs potentially involved in the physiology of the lactating mammary gland. Our study provided further insight into the miRNA-mediated regulation of target genes in lactating mammary gland physiology.

## Supporting Information

Figure S1 **Experimental animals license.**
(TIF)

Figure S2 **The overall flow of sRNA library construction and Solexa sequencing.** 18∼30 nt fraction of total RNA was excised and purified following 15% PAGE. 3′ and 5′ adaptors were respectively ligated using T4 RNA ligase. The adaptor-ligated small RNAs were subjected to RT-PCR amplification, and the cDNA was further amplified. The PCR products were purified and used for sequencing analysis on a Illumina/Solexa Genome Analyzer.
(TIF)

Figure S3 **The bioinformatics analysis flow of Solexa sequencing data.** The image files were converted into raw data by base calling, and the low quality reads were removed. After masking the adaptor sequences and removing contamination, the clean reads were processed for computational analysis. The expression and distribution of clean reads were analyzed by mapping to the *Ovis aries* genome, sequence alignment was done against the known animal miRNAs deposited in the miRBase. The classification annotation of sRNA were analyzed in GenBank and Rfam databases. The unannotated sRNAs were used to predict the presumptive novel miRNA candidates.
(TIF)

Figure S4 **Hierarchical clustering analysis of miRNAs in two samples.** Red indicates the miRNA with high expression in late lactation, green indicates the miRNA with high expression in peak lactation, and gray indicates the miRNA with no expression in at least one sample, the different colors represent the different fold changes (Log2 Late lactation/Peak lactation). The hierarchical clustering of miRNA expression was performed using PermutMatrix software with Pearson distance. All differentially expressed miRNAs were clustered in one by six cluster.
(PNG)

Table S1 **miRNA expression profiles in peak and late lactation.**
(XLS)

Table S2 **Characteristics of novel miRNA candidates.**
(XLS)

Table S3 **Target genes for the differentially expressed miRNAs in peak lactation and late lactation predicted using MIREAPv0.2.** From left to right for each miRNA: miRNA name, miRNA length, target name, target length, match position, MFE, P-value, prediction value.
(XLS)

Table S4 **KEGG Pathway annotations for the predicted target genes.**
(XLS)

Table S5 **GO annotations for the predicted target genes.**
(XLS)
