# Correction: Identification of Novel and Differentially Expressed MicroRNAs of Dairy Goat Mammary Gland Tissues Using Solexa Sequencing and Bioinformatics

**DOI:** 10.1371/annotation/25b72ec2-dcf3-4ac3-b08e-26cf82a61efa

**Published:** 2013-02-04

**Authors:** Zhibin Ji, Guizhi Wang, Zhijing Xie, Jianmin Wang, Chunlan Zhang, Fei Dong, Cunxian Chen

This article reports an analysis of microRNA expression on tissues collected in peak lactation (90 day postpartum) and late lactation (210 day postpartum). The experiments reported in this article are related to and originate from the same animals as those described in our previous publication below:

Identification and characterization of microRNA in the dairy goat (Capra hircus) mammary gland by Solexa deep-sequencing technology.

Ji Z, Wang G, Xie Z, Zhang C, Wang J.

Mol Biol Rep. 2012 Oct;39(10):9361-71.

The Molecular Biology Reports article focused on the analysis of miRNA expression on goat tissues collected in early lactation (20 day postpartum). The publication in Molecular Biology Reports should have been cited in the PLOS ONE article and the authors apologize for this omission.

In addition, in the light of the previous publication in Molecular Biology Reports, the following claims outlined in the PLOS ONE article regarding the originality of the global miRNA expression profile analysis in Capra hircus are inaccurate:

Abstract: 'This study provided the first global of the microRNA in Capra hircus and expanded the repertoire of microRNAs'

Introduction 'In this study, we provided the first global of the miRNA expression profiles (miRNome) in Capra hircus and expanded the repertoire of miRNAs.'

Conclusions 'The present study is the first to examine the goat mammary gland miRNA expression profile' 

