# Supplementary figures and images for: Identification of Novel and Differentially Expressed MicroRNAs of Dairy Goat Mammary Gland Tissues Using Solexa Sequencing and Bioinformatics
Source: PLoS One. 2012 Nov 14;7(11):e49463. doi: 10.1371/journal.pone.0049463 (PMC3498112; doi:10.1371/journal.pone.0049463)

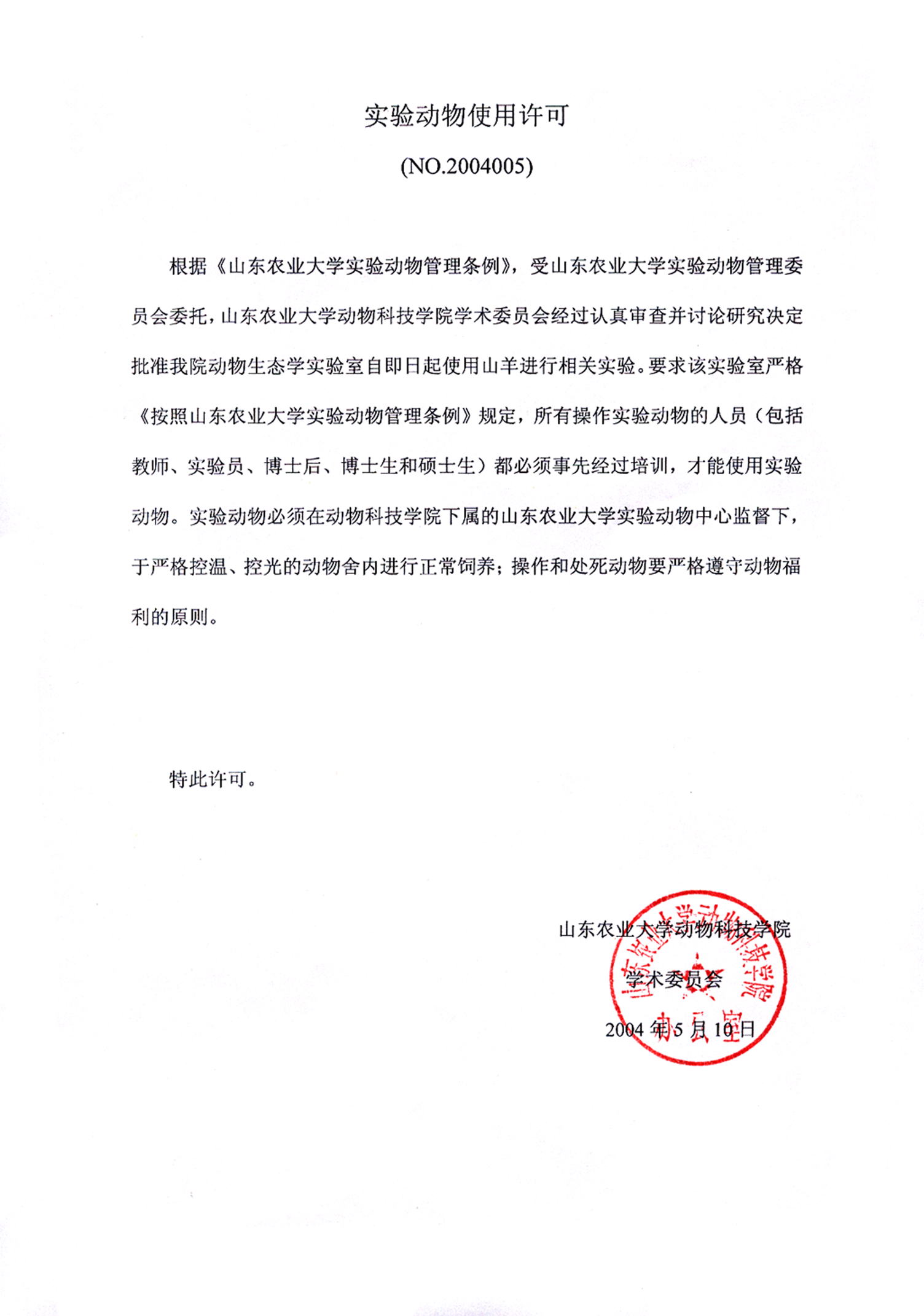

Supplement: Figure S1 — Experimental animals license. (TIF) [file pone.0049463.s001.tif]

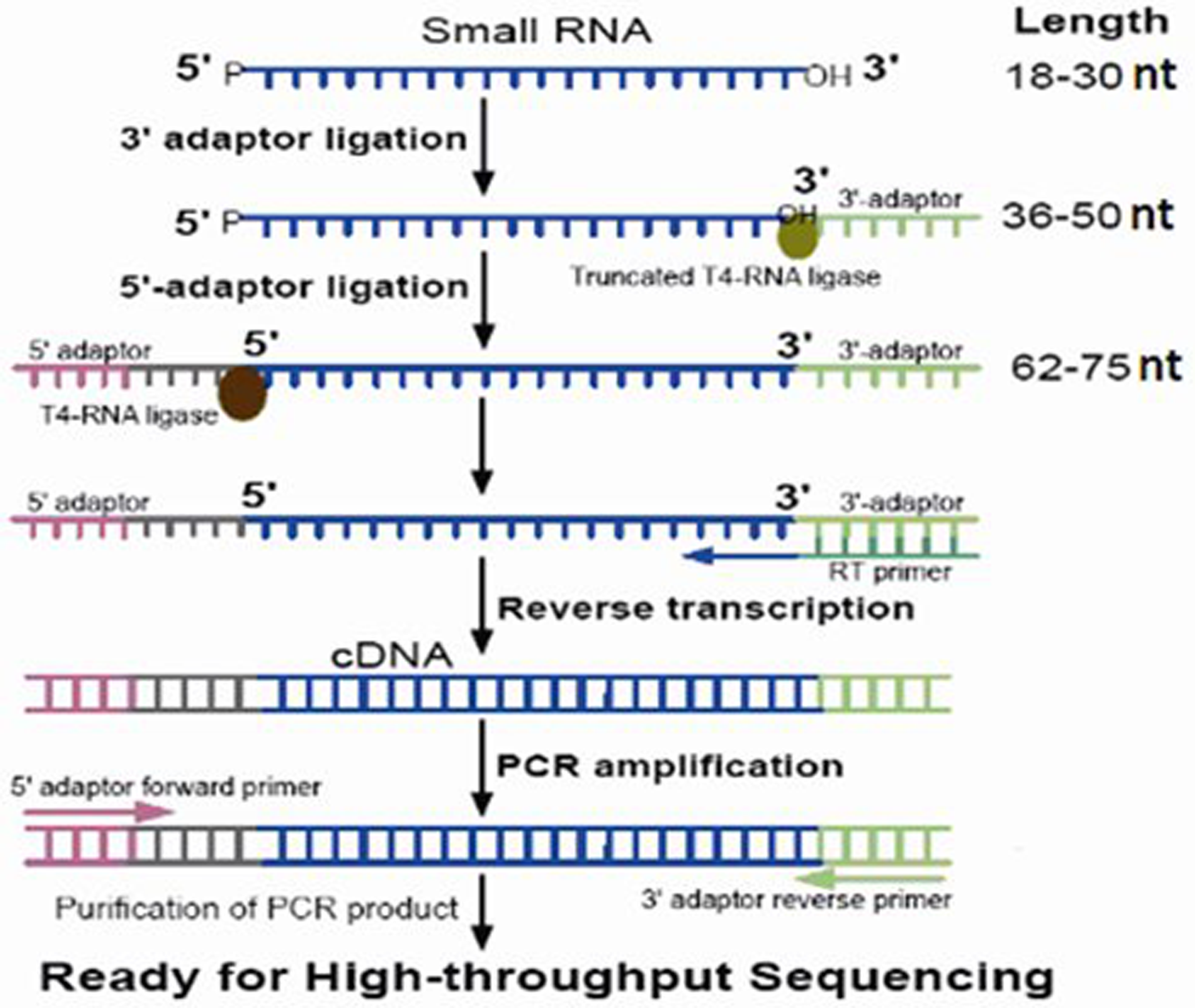

Supplement: Figure S2 — The overall flow of sRNA library construction and Solexa sequencing. 18∼30 nt fraction of total RNA was excised and purified following 15% PAGE. 3′ and 5′ adaptors were respectively ligated using T4 RNA ligase. The adaptor-ligated small RNAs were subjected to RT-PCR amplification, and the cDNA was further amplified. The PCR products were purified and used for sequencing analysis on a Illumina/Solexa Genome Analyzer. (TIF) [file pone.0049463.s002.tif]

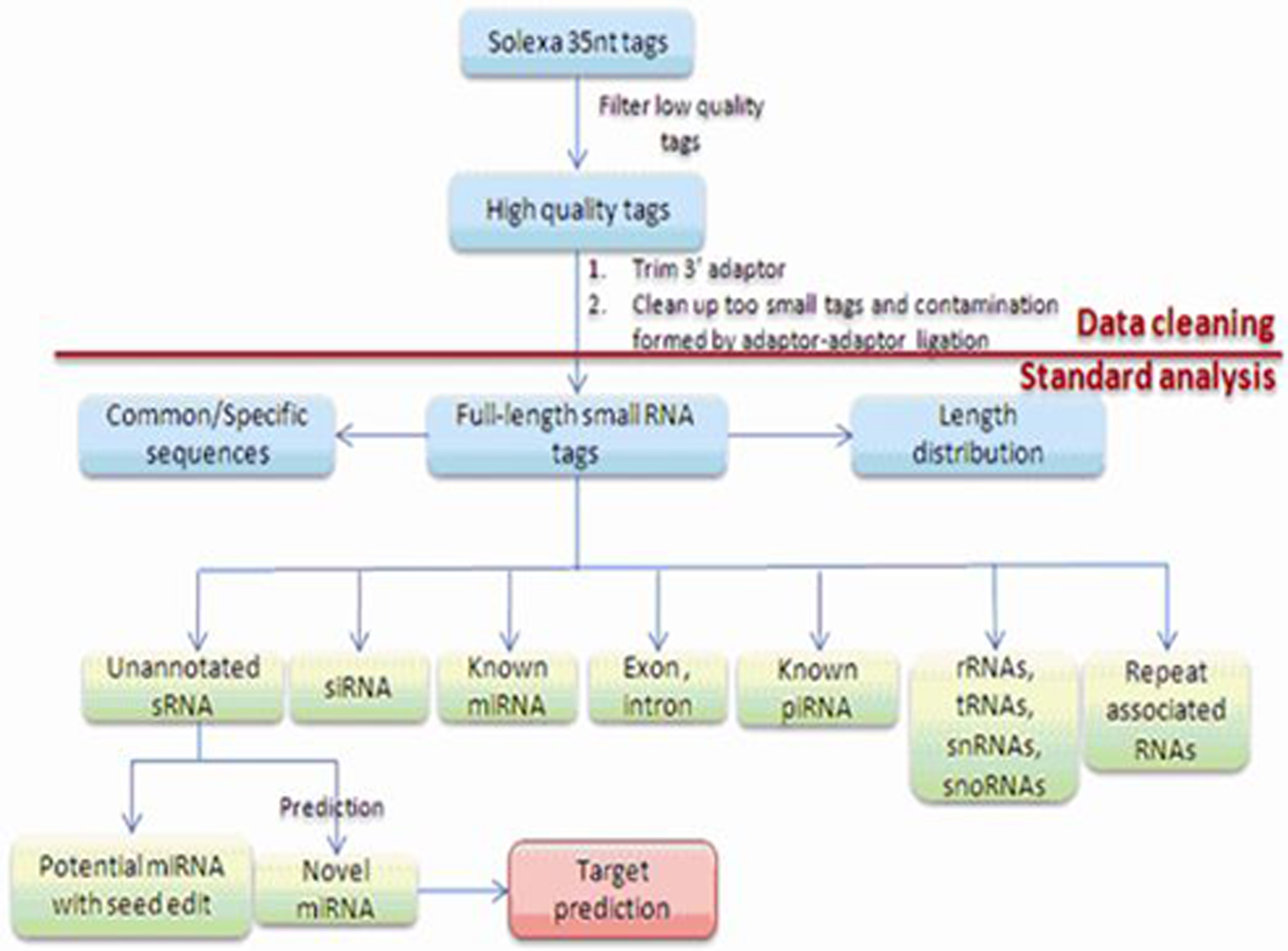

Supplement: Figure S3 — The bioinformatics analysis flow of Solexa sequencing data. The image files were converted into raw data by base calling, and the low quality reads were removed. After masking the adaptor sequences and removing contamination, the clean reads were processed for computational analysis. The expression and distribution of clean reads were analyzed by mapping to the Ovis aries genome, sequence alignment was done against the known animal miRNAs deposited in the miRBase. The classification annotation of sRNA were analyzed in GenBank and Rfam databases. The unannotated sRNAs were used to predict the presumptive novel miRNA candidates. (TIF) [file pone.0049463.s003.tif]
